# Acute and Late Toxicities of Concurrent Chemoradiotherapy for Locally-Advanced Non-Small Cell Lung Cancer

**DOI:** 10.3390/cancers9090120

**Published:** 2017-09-08

**Authors:** Vivek Verma, Charles B. Simone, Maria Werner-Wasik

**Affiliations:** 1 Department of Radiation Oncology, University of Nebraska Medical Center, Omaha, NE 68106, USA; vivek333@gmail.com; 2 Department of Radiation Oncology, University of Maryland Medical Center, Baltimore, MD 21201, USA; charlessimone@umm.edu; 3 Department of Radiation Oncology, Thomas Jefferson University Hospital, Philadelphia, PA 19107, USA

**Keywords:** non-small cell lung cancer, chemoradiotherapy, toxicity, esophagitis, pneumonitis, pulmonary fibrosis, hematologic toxicity, cardiac toxicity

## Abstract

For patients with unresectable locally-advanced non-small cell lung cancer (LA-NSCLC), concurrent chemoradiotherapy improves overall survival as compared to sequential chemotherapy and radiation therapy, but is associated with higher rates of toxicities. Acute, clinically significant esophagitis or pneumonitis can occur in one in five patients. The risks of esophagitis and pneumonitis can impact the decision to deliver concurrent therapy and limit the total dose of radiation therapy that is delivered. Hematologic toxicities and emesis are common toxicities from systemic therapies for LA-NSCLC and can result in delaying chemotherapy dosing or chemotherapy dose reductions. Late treatment morbidities, including pulmonary fibrosis and cardiac toxicities, can also significantly impact quality of life and potentially even survival. Recent advances in radiation therapy treatment delivery, better knowledge of normal tissue radiotherapy tolerances and more widespread and improved uses of supportive care and medical management of systemic therapy toxicities have improved the therapeutic ratio and reduced the rates of chemoradiotherapy-induced toxicities. This review details the acute and late toxicities associated with definitive chemoradiotherapy for LA-NSCLC and discusses toxicity management and strategies to mitigate the risks of treatment-related toxicities.

## 1. Introduction

The second most common noncutaneous neoplasm in both men and women, lung cancer, will be diagnosed in an estimated 222,500 patients in the United States in 2017; it is the most common cause of cancer death in both sexes (155,870 deaths in 2017) [1]. Although a rise in early-stage disease is expected in the future [2,3,4,5], the most common presentation among non-metastatic patients remains locally-advanced non-small cell lung cancer (LA-NSCLC) [6].

The standard of care to manage LA-NSCLC was influenced in great part by the Radiation Therapy Oncology Group (RTOG) 94-10 trial [7]. This trial randomized 610 patients into three arms. The first arm consisted of sequential vinblastine plus cisplatin followed by radiotherapy (RT) to a dose of 63 Gy delivered in 34 daily fractions (sequential chemoradiotherapy (sCRT)). The second arm involved concurrent CRT with the same aforementioned chemotherapy agents and RT dose and fractionation (concurrent chemoradiotherapy (cCRT)). The third arm also consisted of cCRT, but with cisplatin plus etoposide and hyperfractionated RT (69.6 Gy in 58 twice-daily fractions of 1.2 Gy each). The major impact of the trial was the finding of increased overall survival (OS) in the cCRT arm with once-daily RT (17.0 months) over the sCRT arm (14.6 months). Importantly, the study detailed substantial treatment-induced morbidities, including increased toxicities in both cCRT arms.

As such, the focus of this article is to review several common toxicities of cCRT in patients with LA-NSCLC. Specifically, the acute toxicities of esophagitis and radiation pneumonitis will be discussed in addition to chemotherapy-specific adverse events. Late cardiac toxicities and radiation fibrosis are also detailed. Considerations in management of these toxicities and strategies to decrease their frequency and severity are also outlined.

## 2. Acute Toxicities

### 2.1. Esophagitis

The esophagus harbors a relatively rapidly-proliferating squamous epithelium and courses longitudinally through the mediastinum. Owing to anatomic proximity, the esophagus may become inflamed from RT. The RTOG and the Common Toxicity Criteria for Adverse Events (CTCAE, Version 4) scales for acute radiation esophagitis are given in Table 1.

First, chemotherapy exerts independent and potentially synergistic effects on the esophagus. As a result, the receipt of cCRT amplifies esophageal toxicities over singular treatment with either modality alone. Indeed, the RTOG 94-10 trial demonstrated the rate of grade ≥3 esophagitis to be only 4% in the sequential arm, but 22% in the cCRT once-daily RT arm. Additionally, the increase to 45% in the cCRT twice-daily RT arm is consistent with radiobiological principles that hyperfractionation may produce worse acute toxicities in exchange for potential decreased risks of chronic adverse events. In fact, the 22% rate of grade ≥3 esophagitis is a relatively higher quoted value in comparison to other prospective data, wherein an estimated 13% of patients suffer grades ≥3 esophagitis [8] and notably higher than 4% of sequentially-treated patients in a meta-analysis of cCRT versus sCRT trials [9].

Next, symptomatically, radiation esophagitis most commonly presents with dysphagia, odynophagia and/or reflux-like symptoms including epigastric and sternal chest pain. Symptoms may be exacerbated by preexisting esophageal disease (e.g., gastroesophageal reflux). The presence of esophageal bleeding or perforation is rare, especially without preexisting esophageal pathology. Radiation esophagitis should be suspected in a patient with symptomatology who is undergoing thoracic radiotherapy with appreciable doses to the mediastinum, especially with large-volume and/or centrally-located disease that is in close proximity to the esophagus. 

Management revolves around symptomatic control, including topical anesthetics (e.g., viscous lidocaine), pain medications (non-steroidal anti-inflammatory drugs and/or opioids) and proton pump inhibitors (if applicable). Persistent symptoms may necessitate a trial of antifungal medications, as candidiasis is not uncommon in relatively immunocompromised patients receiving cCRT. If symptoms are particularly severe, treatment breaks may be required, but treatment delays should be avoided whenever possible in efforts to avoid prolonging the overall RT treatment time and allowing for tumor repopulation, which can have an impact on clinical outcomes [10,11]. Chemotherapy reductions may also provide a benefit, although if the esophagus is receiving high RT doses, there may be relatively less impact of modifying chemotherapy in order to decrease symptoms. Accordingly, the RTOG 98-01 study evaluated the radioprotector amifostine for the reduction of esophagitis in the hyperfractionated RT setting, but did not observe differences in grades ≥3 esophagitis [12].

In sum, in order to minimize the risk of developing (or the severity of) esophagitis, particularly severe esophagitis, RT treatment planning should respect dose constraints of the esophagus. There have been many studies demonstrating correlations between esophagitis and radiation doses delivered to various volumes of the esophagus (termed dose-volume parameters) [13,14,15,16,17,18,19]. Although different studies have implicated several different parameters, some recommended constraints include mean esophageal dose <34 Gy and/or the volumes of esophagus receiving at least 35, 50 and 70 Gy to be <50%, <40% and <20%, respectively [20].

### 2.2. Radiation Pneumonitis

Radiation pneumonitis (RP) occurs as a result of the sensitivity of normal lung parenchyma to RT. Acute RP rarely occurs during treatment and instead most commonly manifests in the first six months after RT completion [21]. However, progression may occur to encompass chronic pulmonary damage beyond 3–6 months. Thus, RP is a substantial concern during RT treatment planning of LA-NSCLC, especially as the volume of disease increases. Whereas radiation esophagitis rarely leads to death, RP certainly can do so, with a ~2% rate of fatal RP [21,22], although modern treatment planning techniques lower this risk further. Grade ≥3 acute pulmonary toxicities occurred in 9%, 4% and 2% in the sCRT, cCRT (daily RT) and cCRT (twice daily RT) groups, respectively, in RTOG 94-10 [7].

Next, the mechanisms of RP provide an important basis for treatment options [23]. As early as hours after RT exposure to the lungs, hallmarks of acute inflammation predominate, including hyperemia, increased capillary permeability, leukocytic infiltration and cytokine release (e.g., interleukin-6, interleukin-1α and tumor necrosis factor-α). Days to weeks thereafter, exudative alveolitis and sustained acute inflammation leads to the symptomatic phase, which is characterized by pathological sloughing of type I pneumocytes and endothelial cells. Corresponding symptoms include a nonproductive cough, presence or worsening of dyspnea, fever or chills, constitutional symptoms such as malaise and weight loss and chest pain (most commonly pleuritic in nature). Distinction must be made, initially using history and physical examination, between RP, infectious pneumonia, chronic obstructive pulmonary disease exacerbation and tumor progression or the mass effect of the primary neoplasm on lung parenchyma and/or airways. For a clinical diagnosis, RP identification may be greatly assisted through imaging, especially if opacities on chest radiographs and/or computed tomography (CT) scans are present in a straight line following the course of the radiation portal that is typically delivered for 3D conformal radiation therapy.

Consequently, the cornerstone of management of RP is glucocorticoids, along with supplemental oxygen if clinically indicated. Notably, treatment of RP correlates with the grading of its severity (Table 2). It is also important to recognize the lack of randomized trials comparing the efficacy of various interventions and agents, including corticosteroids. There have been studies of several other drugs such as pentoxifylline [24] and amifostine [25] that have also produced low incidences of RP, but have not been tested against corticosteroids for RP in a phase III manner. 

Additionally, preventative measures are an important aspect of management. Substantial effort during RT treatment planning is spent in order to ensure that several dose-volume parameters are within tolerance limits so as to minimize the risk of clinical RP. The two most commonly-utilized parameters are the mean lung dose (MLD) and the normal bilateral lung volume (generally minus the tumor volume) receiving at least 20 Gy (V20) [26,27,28]. Depending on the particular study, attempting to keep the V20 ≤ 30–37% and/or the MLD ≤ 20 Gy results in an estimated RP rate of 20% or less [22,26,27]. It should also be noted that there is significant heterogeneity among LA-NSCLC patients in terms of RP risk, with factors such as smoking history and current smoking, patient age, pre-existing pulmonary function, tumor size and location, thoracic anatomy and even gender and marital status all having been associated with the risk of developing RP [28,29,30,31,32,33]. 

Lastly, there are two important factors that have recently come to the forefront of investigation as to potential correlations with RP. First, newer RT techniques (to be discussed subsequently) often increase the number of radiation beams directed to the tumor volume. Although such approaches increase the conformality and allow for reduced higher-dose spillage to surrounding lung, it results in higher lung volumes receiving lower irradiation doses. In light of this, one series of 223 patients found V5 (volume of normal lungs receiving ≥5 Gy) as the only parameter associated with RP [34]. Although much less reproduced by other clinical reports than MLD or V20, this parameter is increasingly being taken into account during treatment planning and must be further studied as advanced RT techniques become more ubiquitous. Next, it has been recently reported in an international cohort of 836 patients with LA-NSCLC that receipt of carboplatin and paclitaxel chemotherapy, especially in patients over 65 years of age, was associated with an independently higher risk of RP [22]. However, other data have not supported this notion [35], and this drug regimen was not as widely used as cisplatin and etoposide-based combinations used in trials such as RTOG 94-10 that did not show an increase in RP with cCRT. Nevertheless, the data are compelling and require prospective data assessing the tradeoffs between the relative ease and convenience of carboplatin and paclitaxel administration relative to a potentially higher RP incidence. 

### 2.3. Hematologic Toxicities

Because chemotherapy is a form of systemic therapy, it carries risks of separate toxicities. Principally, hematologic toxicities (granulocytopenia, anemia, thrombocytopenia and leukopenia) can compromise the receipt of further chemotherapy (or RT) and hence may impact outcomes. In RTOG 94-10, the overall rate of grade ≥3 thrombocytopenia, leukopenia and granulocytopenia was 10%, 70% and 71%, respectively [7]. However, only 3% of patients had major protocol deviations from chemotherapy. Nausea and emesis can also often occur from chemotherapy; additionally, anticipatory nausea prior to chemotherapy occurs in approximately one in 10 patients [36]. Although antiemetics most often result in relief of symptoms, nutritional monitoring in this population is quite important in order to ensure adequate oral intake.

From the radiotherapy perspective, nausea and emesis may also occur in patients with left lower lobe disease who receive appreciable gastric irradiation doses, or with right lower lobe disease who receive appreciable hepatic irradiation doses as a result of anatomic proximity. Premedication with antiemetics may help select patients. Additionally, RT is not thought to be as appreciably associated with hematologic toxicities, although irradiation doses to vertebral bone marrow have been understudied in lung cancer to date [37]. However, the significance of neutropenia is its strong association with RT-induced dysphagia/esophagitis [38]. Mechanistic hypotheses include neutrophils being necessary to protect/heal the esophageal mucosa, or being a surrogate for bone marrow stem cells that repair the esophageal damage. Regardless, aside from chemotherapy breaks and dose reductions, select patients may benefit from blood cell precursor colony-stimulating factors.

## 3. Late Toxicities

### 3.1. Cardiotoxicity

Historically, minimizing cardiac irradiation doses has not been prioritized to a great degree during treatment of LA-NSCLC, largely owing to the understanding that cardiac adverse events could take years or even decades to develop and generally poor prognosis and low life expectancy of this population [39]. This is in stark contrast to the relative wealth of data for malignancies with good prognoses (e.g., pediatric, Hodgkin lymphoma and breast cancers) that have correlated heart doses with cardiac events [40,41,42]. Thirteen total patients of 531 (2.4%) were recorded as having grade ≥3 heart toxicities in RTOG 94-10 [7], although it is admittedly difficult to capture individual cardiac events (whether attributable to RT or other causes) throughout patient follow-up. 

**Figure 1:**
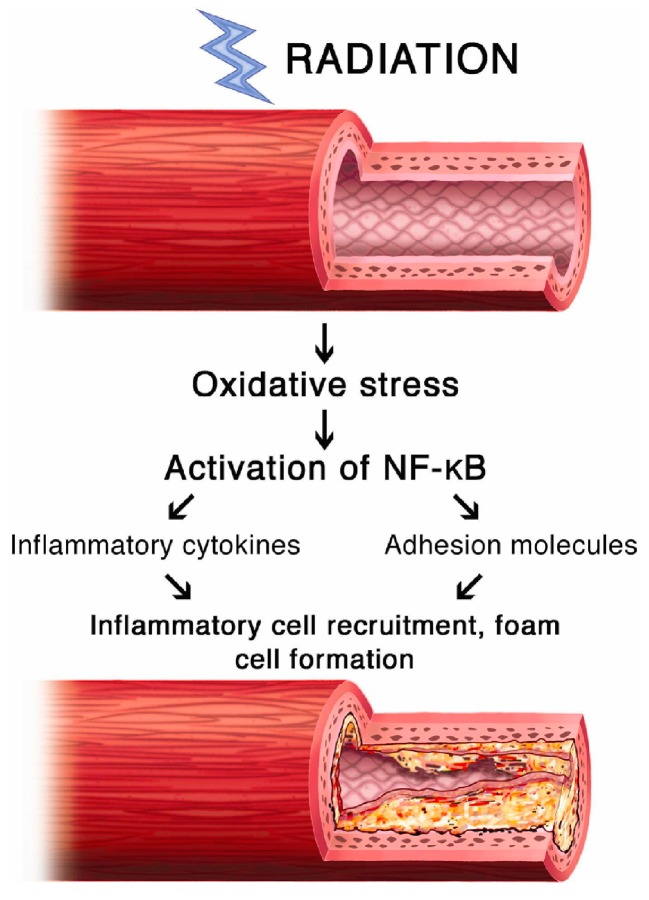
Mechanisms of radiation-induced cardiovascular injury. Utilized with permission from Weintraub et al. [44].

There are many forms of RT-induced cardiac toxicities. Radiation can affect the pericardium, muscle, the electrical conduction system, valves and vasculature, among other structures. Mechanistically, the effects of RT on vasculature may be most important for pathophysiologic correlations of heart toxicity (Figure 1). The effects of RT on small vessels, regardless of location, are that of fibrosis (possibly preceded by some level of chronic inflammation) and endothelial injury (potentially a partially obliterative endarteritis phenotype). This not only can predispose to arterial disease from preexisting causes (e.g., lipid dysregulation and atherosclerosis), but is also an independent risk factor for chronic ischemia and future cardiac events.

With regard to NSCLC, although cardiac toxicity has largely been understudied, population-based analyses have demonstrated independently increased risks of cardiac pathologies in patients that received RT, chemotherapy or CRT [43]. There is evidence of a dose-dependent relationship, as left-sided (but not right-sided) cancers were independently associated with several cardiac pathologies on multivariate analysis. Intuitively, patients at higher risk of cardiac toxicities included those with comorbidities, advanced age and disease, as well as demographic factors associated with heart disease (e.g., male gender and black race). Whether cCRT is associated with an increased risk of cardiac adverse effects over sCRT or either modality alone is currently unknown. 

Consequently, cardiac toxicity in LA-NSCLC has recently come to the forefront of NSCLC radiation therapy in part because of novel results from the recently reported RTOG 0617 trial [45]. This study was a phase III trial randomizing patients to 60 Gy versus 74 Gy of RT as part of cCRT in the definitive setting, with the major finding that the high-dose arm had statistically poorer survival. Moreover, the authors illustrated that independent predictors of OS (on multivariate analysis) included cardiac irradiation doses, including heart V5 and V30. Importantly, heart doses were statistically higher in the high-dose arm. Although dose constraints were given in the protocol, compliance was not mandated. These findings bring forth several questions. First, perhaps the dogma of “late cardiac mortality” being relatively trivial for LA-NSCLC should be re-assessed. Moreover, it is possible that acute effects of (larger) cardiac doses may contribute to excess mortality.

Taken together, further work is needed to shed light on these and other radiation-induced cardiotoxicity issues. For instance, although a retrospective institutional study of 468 patients could not corroborate the results of cardiac dosimetry on OS as in RTOG 0617, the authors noted that mean heart dose and heart V5 correlated with MLD, the latter of which was the only significant dosimetric predictor of OS [46]. Another caveat to the measurement of cardiac doses and mortality was pointed out by investigators at Wake Forest University; although noticing an association with cardiac mortality and RT usage in NSCLC patients, the RT-induced risk of heart disease decreased in the modern era [47]. This may be due to advancements in radiation techniques and technologies, which is the focus of a subsequent section. Despite this, most recently, in a large single-institution retrospective analysis from Washington University, investigators found that heart dose was an independent predictor of overall survival for LA-NSCLC [48].

### 3.2. Pulmonary Fibrosis

Whereas acute lung injury was discussed previously, chronic parenchymal damage manifests as scarring and is seen relatively commonly on post-treatment imaging as part of routine surveillance follow-up. Although many cases are asymptomatic, symptomatic cases often involve high chronic inflammation characterized by high levels of circulating platelet-derived and basic fibroblast growth factor expressed after initial acute inflammation. This leads to fibroblast proliferation and migration, the release of a major pro-fibrotic molecule known as transforming growth factor-β, along with collagen deposition. Collagen may be deposited in any histologic space of the irradiated lung, including vascular and alveolar compartments; this can lead to ventilation-perfusion mismatch and result in worsening of pulmonary function (or even functional status) as a primary symptom [23]. This may also be corroborated by imaging to a similar extent of linear consolidation following the track of the radiation portal. Other symptoms may be similar to acute RP, including nonproductive cough and dyspnea, although these symptoms are generally more chronic in nature. Owing to the pathophysiologic time course, symptoms are not seen until several months after RT and may continue to progress for years after therapy. 

Diagnosing pulmonary fibrosis can often be challenging. The differential diagnosis often involves infectious causes (especially in the case of fever, which is uncommon in isolated radiation fibrosis), worsening of chronic obstructive pulmonary disease and tumor progression. Grading (Table 3) involves assessment of imaging, symptoms and interventions (if applicable). The RTOG 94-10 trial demonstrated similar numbers of late grade ≥3 pulmonary toxicities in all three arms (15%, 13% and 17% in the sCRT, cCRT with daily RT and cCRT with twice-daily RT, respectively) [7]. 

Treatment, similar to RP, centers on corticosteroids and/or symptomatic management. Furthermore, involvement of pulmonary providers in addition to the oncologic team, with cardiology input when necessary, is necessary for optimal management (especially in the acute setting). Interventions such as pentoxifylline, vitamin E and/or hyperbaric oxygen have limited data supporting their use and are usually considered on a case-by-case basis. However, some small studies have demonstrated treatment efficacy with such agents, bringing to question whether pulmonary fibrosis is truly an irreversible process as generally thought [49]. Nevertheless, the most important management aspect of radiation fibrosis is prevention using several dose-volume parameters discussed previously, such as MLD and V20, among others [50]. Although acute RP is typically thought to be more dose limiting and life threatening, further work is needed in radiation fibrosis to better define dose-volume parameters and subpopulations in which stricter constraints are necessary.

## 4. Strategies to Reduce Toxicities

Toxicities of cCRT may arise to different degrees in various subpopulations and scenarios. First, with regards to chemotherapy, the presence of important variables such as age, performance status, and pre-existing comorbidities plays a large role in selecting patients fit to tolerate cCRT. Select patients may also benefit from chemotherapy alterations in doses, intervals or even specific compounds and regimens. If toxicities persist, sCRT may be an option since the receipt of bimodality therapy in any capacity is often preferred to single-agent therapy alone if concurrent administration is contraindicated or not tolerated, although based on RTOG 94-10, such an approach is associated with inferior overall survival compared with cCRT [7].

Second, from the RT perspective, there are many reasons to believe that toxicities are reduced from greater utilization of new RT technologies as compared to historical trials of cCRT. First, image-guided RT has now become the standard of care in any center with such capabilities, potentially allowing for reduced target volume margins that may prove to decrease toxicities. Next, the RTOG 0617 trial has set the “standard” RT dose for LA-NSCLC to 60 Gy. With the knowledge of decreased survival (possibly due to greater rates of treatment-related toxicities) in patients treated to 74 Gy, toxicities may be reduced with practice patterns shifting to lower doses. 

Consequently, because advanced radiotherapy techniques have developed in the years after the publication of seminal trials of CRT for NSCLC, there is reason to believe that utilization of these techniques may result in fewer observed toxicities in the modern era. For instance, intensity-modulated RT (IMRT) further decreases doses to nearby organs-at-risk over techniques used in prior trials by utilizing inverse-planned computer algorithms to create highly conformal dose distributions. IMRT is well known to offer clinically superior toxicity profiles over three-dimensional forward planning for several tumor types, most prominently those that involve close apposition of target volumes and organs at risk [51]. Thus, when performing concurrent CRT, highly conformal RT techniques such as IMRT can be considered to attempt to reduce the volume of irradiated normal lung, heart and esophagus [52]. In fact, two interesting secondary analyses of RTOG 0617 have been recently published. In the first, IMRT was associated with better preservation of quality of life compared with 3DCRT [53]. In the second, despite IMRT being more commonly delivered to patients with more advanced disease and larger target volumes in the trial, IMRT was associated with less grade ≥3 pneumonitis (*p* = 0.039) and lower heart doses (*p* < 0.05) [54].

Additionally, proton beam therapy (PBT) has also emerged as a potential option for treating lung cancer [55,56,57], which when delivered using advanced techniques and approaches (e.g., pencil-beam scanned PBT or intensity-modulated proton therapy) [58], can further reduce dose to normal tissues compared with photon therapy [59,60]. A fundamental principle of PBT is the ability to place the location of maximal dose deposition (termed the Bragg peak) and provide essentially no exit dose distal to the target of interest. Doing so decreases the overall integral dose to patients and decreased volumetric doses to surrounding organs-at-risk. However, a substantial knowledge gap exists as to whether dosimetric gains translate into clinically-reduced toxicities and, if so, in which subgroups of LA-NSCLC PBT will be most useful. In fact, the results presented of a phase II randomized trial did not observe differences in rates of RP [61]. Other issues associated with PBT need convincing resolution, as well, including range uncertainties and plan robustness relating to tissue heterogeneity in the beam path, as well as economic sustainability and cost effectiveness of PBT [62,63]. The ongoing RTOG 1308 phase III study is seeking to compare OS in stage II–IIIB patients treated with PBT versus photon RT [64]. When mature, this trial will provide the highest level of evidence to date determining whether PBT can enhance the therapeutic ratio over photon-based RT, along with several secondary endpoints such as quality of life and cost effectiveness. Results of these trials will have major implications and may lead to a reevaluation of the therapeutic ratio for LA-NSCLC.

Lastly, the value of proper supportive care cannot be understated in any form of oncologic therapy. This extends not only to individuals close to the patient, but also hospital-based teams. With regards to a multidisciplinary oncologic care team, the assistance of social workers, nurses, psychologists, counselors, therapists and nutritionists, among others, is essential. All members of the oncologic team should ideally be able to recognize common toxicities and conditions (e.g., depression) in all patients, including early identification of those patients that are at higher risk of developing toxicities, and respond with early and effective interventions. With respect to oncologic and lung cancer patients, careful patient selection for nutritional support and intravenous hydration can help to reduce the patient experiences of therapy-induced adverse effects [65,66].

## 5. Conclusions and Future Directions

Over the past decade, molecular oncology has rapidly elucidated so-called “driver mutations” for NSCLC that have led to the emergence of targeted therapies. Standard treatment for epidermal growth factor receptor (EGFR) and anaplastic lymphoma kinase (ALK) mutant NSCLC now involves biologic therapies [67]. Additionally, several clinical trials are examining the efficacy of these treatments for several mutation-based NSCLC, such as BRAF and HER2, among several others [68]. The attraction of these compounds in limiting toxicities lies with the associated “molecular precision” of these agents, as opposed to the more nonspecific nature of most existing chemotherapeutic formulations. While these agents have primarily been used in the metastatic NSCLC to date, their addition prior to concurrent CRT is currently being tested in RTOG 1306. 

Additionally, the relationship between RT and the immune system is now being actively investigated. RT induces both immune and inflammatory responses, the molecular and genetic mechanisms for which are beginning to be elucidated, which have implications for creating novel biomarkers for response to RT through activation of the immune system [69]. Another major active area of contemporary interest includes the systemic, immune-related response to RT and CRT. These may affect the perceived and/or measured toxicity of therapy and can lead to the phenomena known as the abscopal and bystander effects, which result in tumor and/or normal tissue damage outside of the irradiated field [70]. As a result, maximizing the synergy of the immune response may prove beneficial for the RT-induced tumor response, and strategies to reduce inflammation may help to mitigate RT-induced normal tissue toxicities [71].

Similarly, the concept of immunotherapy, utilizing compounds to drive oncogenic antigen expression and augmenting the de novo immune system to attack existing disease, has been rapidly gaining momentum in the stage IV setting and has emerged as a standard of care for patients progressing on first line chemotherapy [72,73] and more recently in select patients with newly-diagnosed and previously untreated metastatic disease [74]. Like targeted therapies, immunotherapies have lower rates of toxicities compared with cytotoxic chemotherapies. Only recently have immunotherapies begun to be tested in non-metastatic NSCLC [75]. Combining RT with targeted therapy and immunotherapies for LA-NSCLC is a novel approach for attempting to improve outcomes in patients [76,77]. Toxicity profiles of combining these drugs with chemoradiation are not well described to date, and much investigation into the optimal timing and sequencing of these agents is needed.

## Figures and Tables

**Table 1:** Commonly-utilized esophagitis grading criteria.

Scale	Grade 1	Grade 2	Grade 3	Grade 4	Grade 5
RTOG	Mild dysphagia or odynophagia; may require topical anesthetic, non-narcotic agents, or soft diet	Moderate dysphagia or odynophagia; may require narcotics agents or puree/liquid diet	Severe dysphagia or odynophagia with dehydration or weight loss (>15% from pretreatment baseline) requiring nasogastric feeding tube, IV fluids, or hyperalimentation	Complete obstruction, ulceration, perforation or fistula	Death from esophagitis or complications
CTCAE	Asymptomatic; clinical or diagnostic observations only; intervention not indicated	Symptomatic; altered eating/swallowing; oral supplements indicated	Severely altered eating/swallowing; tube feeding, TPN or hospitalization indicated	Life-threatening consequences; urgent operative intervention indicated	Death

Abbreviations: RTOG, Radiation Therapy Oncology Group; CTCAE, Common Toxicity Criteria for Adverse Events (Version 4.0); IV, intravenous; TPN, total parenteral nutrition.

**Table 2:** Commonly-utilized acute pneumonitis grading criteria.

Scale	Grade 1	Grade 2	Grade 3	Grade 4	Grade 5
RTOG	Mild symptoms of dry cough or dyspnea on exertion	Persistent cough requiring narcotic, antitussive agents/dyspnea with minimal effort but not at rest	Severe cough unresponsive to narcotic antitussive agent or dyspnea at rest/clinical or radiological evidence of acute pneumonitis/intermittent oxygen or steroids may be required	Severe respiratory insufficiency/continuous oxygen or assisted ventilation	Death
CTCAE	Asymptomatic; clinical or diagnostic observations only; intervention not indicated	Symptomatic; medical intervention indicated; limiting instrumental ADL	Severe symptoms; limiting self-care ADL; oxygen indicated	Life-threatening respiratory compromise; urgent intervention indicated (e.g., tracheotomy or intubation)	Death

Abbreviations: RTOG, Radiation Therapy Oncology Group; CTCAE, Common Toxicity Criteria for Adverse Events (Version 4.0); ADL, activities of daily living.

**Table 3:** Commonly-utilized pulmonary fibrosis grading criteria.

Scale	Grade 1	Grade 2	Grade 3	Grade 4	Grade 5
RTOG	Asymptomatic or mild symptoms (dry cough); slight radiographic appearances	Moderate symptomatic fibrosis or pneumonitis (severe cough); low grade fever; patchy radiographic appearances	Severe symptomatic fibrosis or pneumonitis; dense radiographic changes	Severe respiratory insufficiency/continuous oxygen/assisted ventilation	Death
CTCAE	Mild hypoxemia; radiologic pulmonary fibrosis	Moderate hypoxemia; evidence of pulmonary hypertension; radiographic pulmonary fibrosis 25–50%	Severe hypoxemia; evidence of right-sided heart failure; radiographic pulmonary fibrosis >50–75%	Life-threatening consequences (e.g., hemodynamic/pulmonary complications); intubation with ventilatory support indicated; radiographic pulmonary fibrosis >75% with severe honeycombing	Death

Abbreviations: RTOG, Radiation Therapy Oncology Group; CTCAE, Common Toxicity Criteria for Adverse Events (Version 4.0).
